# First evidence for a multienzyme complex of lipid biosynthesis pathway enzymes in *Cunninghamella bainieri*

**DOI:** 10.1038/s41598-018-21452-4

**Published:** 2018-02-15

**Authors:** Shuwahida Shuib, Izyanti Ibrahim, Mukram Mohamed Mackeen, Colin Ratledge, Aidil Abdul Hamid

**Affiliations:** 1 School of Biosciences and Biotechnology, Faculty of Science and Technology, Universiti Kebangsaan Malaysia, 43600 UKM Bangi, Selangor Malaysia; 2 School of Chemical Sciences and Food Technology, Faculty of Science and Technology, Universiti Kebangsaan Malaysia, 43600 UKM Bangi, Selangor Malaysia; 3 Institute of Systems Biology, Universiti Kebangsaan Malaysia, 43600 UKM Bangi, Selangor Malaysia; 4 Department of Biological Sciences, University of Hull, Kingston upon Hull, HU6 7RX United Kingdom

## Abstract

Malic enzyme (ME) plays a vital role in determining the extent of lipid accumulation in oleaginous fungi being the major provider of NADPH for the activity of fatty acid synthase (FAS). We report here the first direct evidence of the existence of a lipogenic multienzyme complex (the lipid metabolon) involving ME, FAS, ATP: citrate lyase (ACL), acetyl-CoA carboxylase (ACC), pyruvate carboxylase (PC) and malate dehydrogenase (MDH) in *Cunninghamella bainieri* 2A1. Cell-free extracts prepared from cells taken in both growth and lipid accumulation phases were prepared by protoplasting and subjected to Blue Native (BN)-PAGE coupled with liquid chromatography–tandem mass spectrometry (LC-MS/MS). A high molecular mass complex (approx. 3.2 MDa) consisting of the above enzymes was detected during lipid accumulation phase indicating positive evidence of multienzyme complex formation. The complex was not detected in cells during the balanced phase of growth or when lipid accumulation ceased, suggesting that it was transiently formed only during lipogenesis.

## Introduction

Most microbial cells normally contain no more than 10% (w/w) lipid which occurs mainly in cell membranes as well as other membranous structures. However, oleaginous microorganisms have the capability to accumulate more than 20% (w/w) lipid and some can produce up to 70% of their cell biomass as lipid^1^. This lipid is stored as droplets within the cells and is in the form of triacylglycerols. A number of microorganisms, mainly fungi and some protists, produce lipids containing high contents of polyunsaturated fatty acids (PUFAs) such as γ-linolenic acid (GLA), arachidonic acid (ARA), docosahexaenoic acid (DHA) and eicosapentaenoic acid (EPA). Some of these organisms have been commercially developed as sources of PUFAs, with current sales of several thousands of tons per annum^2^.

The regulation of lipid accumulation in oleaginous yeasts and fungi has been well established^3–5^. The initiation of lipid accumulation occurs when the cells that have been growing in a balanced medium are placed under a nutrient stress: usually the exhaustion of nitrogen from the medium. This leads to a cascade of metabolic events which results in cessation of growth, as protein and nucleic acid synthesis is no longer possible, while the excess carbon is continually assimilated by the cells and is preferentially channelled into lipids. When non-oleaginous microorganisms are placed in similar circumstances, polysaccharides may accumulate instead of lipids. Several enzymes have been implicated in the regulation of lipid biosynthesis. These include malic enzyme (ME), fatty acid synthase (FAS) and ATP: citrate lyase (ACL)^6^. However, of paramount importance is ME (EC 1.1.1.40) which catalyses the irreversible decarboxylation of malate to pyruvate with the generation of NADPH. This enzyme has been shown to be important in determining the extent of lipid accumulation^7,8^.

Although other enzymes, such as glucose-6-phosphate dehydrogenase (G-6-PDH), 6-phosphogluconate dehydrogenase (6-PGDH) and NADP^+^: isocitrate dehydrogenase (NADP^+^: ICDH), could contribute to the pool of NADPH, ME has been suggested to play a key role as the sole NADPH provider for fatty acid biosynthesis catalysed by FAS in three oleaginous fungi; *Mucor circinelloides*, *Mortierella alpina*^9^ and *Cunninghamella bainieri* 2A1^10^. (The biochemistry of no other oleaginous fungus has been studied in similar detail). In each of these fungi, the amount of lipid that is accumulated reaches a finite level. In *Mcr circinelloides*, this is about 25%, in *Mta alpina* it is about 50%^9^ and in *C*. *bainieri* it is about 30%^10^. Cessation of lipid accumulation in all three fungi directly correlates with the loss of ME activity even though other NADPH-generating enzymes continue to be active^9,10^.

In *Mcr circinelloides*^11^ and *Rhodotorula glutinis*^12^, substantial increases in lipid content occur when the ME gene coding for ME (EC 1.1.1.40) was cloned and over-expressed. Furthermore, the lipid content of *Mcr circinelloides* decreased significantly from 24 to 2% (w/w) when sesamol (specific inhibitor of ME activity) was added to the cultures^7^. These results have been interpreted as strongly implicating a direct role of ME in lipid biosynthesis in oleaginous fungi.

It has been proposed that the NADPH requirement for FAS reactions in *Mcr circinelloides*, *Mta alpina* and *C*. *bainieri* 2A1 is specifically supplied by ME and not from the general “pool of NADPH”^4,10^. A possible explanation for this is the occurrence of a multienzyme complex involving ME and FAS that would allow direct channelling of NADPH during lipid biosynthesis only from ME but not from other NADPH generating enzymes^5,10^. Enzymes that are involved in sequential reactions in a pathway are often physically associated with each other, forming temporary or stable larger multienzymes complexes^13,14^. This physical association of enzymes involved in the same pathway allows direct channeling of intermediates through the sequence of reactions in the pathway as products generated by each of the enzymes are immediately transferred to the active site of the subsequent enzymes without equilibrating with the global aqueous system where the complexes are located in cells. This allows the working concentration of intermediates of the enzymes to be efficiently achieved. Thus, physical association of the enzymes could increase the efficiency, specificity and speed of metabolic pathways in cells. To date many multienzyme complexes have been elucidated such as the TCA cycle, triacylglycerol (TAG) biosynthesis and respiratory complexes^15–18^.

**Figure 1:**
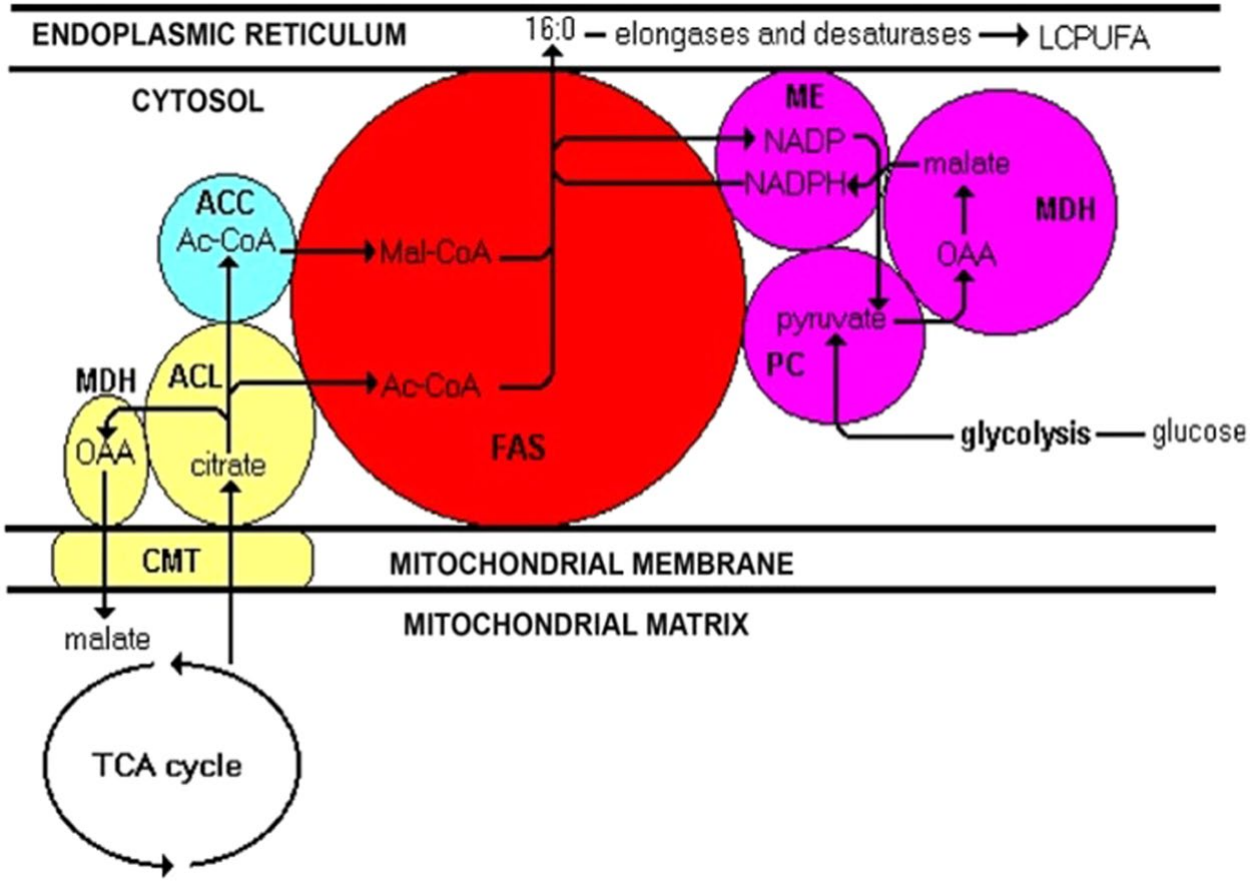
A diagram to show the organization of a hypothesized lipogenic metabolon. The flux of carbon from the mitochondrion, via citrate efflux and acetyl-CoA formation in the cytosol, then into fatty acids and finally into long chain PUFAs (LCPUFA) occurring in the membranes of the endoplasmic reticulum, is shown by the continuous lines. The system uses pyruvate (from glycolysis) as the provider of intramitochondrial acetyl-CoA and for citric acid production as is shown in Fig. 1 but which is not repeated here for clarity. OAA: oxaloacetate; AC-CoA: acetyl-CoA; Mal-CoA: malonyl-CoA; FAS: fatty acid synthase; ACL: ATP: citrate lyase; ACC: acetyl-CoA carboxylase; CMT: citrate/malate translocase; ME: malic enzyme; PC: pyruvate carboxylase; MDH: malate dehydrogenase. For sake of clarity, and in the absence of being able to depict the model in 3 dimensions, it should be noted that, although MDH is depicted in two locations, it is in fact the same protein. The ‘yellow’ enzymes form the citrate/malate cycle while the ‘purple’ enzymes represent the cytosolic “transhydrogenase cycle^5^”.

The existence of a lipogenic multienzyme complex consisting of ME, FAS, ACL, acetyl-CoA carboxylase (ACC), pyruvate carboxylase (PC) and malate dehydrogenase (MDH) has been hypothesized^5^ (as shown in Fig. 1). The hypothesis involves a “transhydrogenase cycle” consisting of PC, MDH and ME that operates independently in the generation of NADPH and the malate-citrate shuttle that generates acetyl-CoA. Both NADPH and acetyl-CoA are subsequently channelled to FAS^8^.

Several reports regarding physical association of enzymes implicated in lipid biosynthesis in non-microbial systems have been reported. The physical association of ACC, ACL and FAS in the microsomal fractions of rat liver, suggesting the microsome as being the major site for the localisation of fatty acid synthesis multienzyme complex^19^. In addition, ME exists in a membrane-bound form in the oleaginous fungus *Mcr circinelloides*^20^ possibly providing NADPH for the fatty acid desaturases to operate. However, to the best of the authors’ knowledge, there has been no report regarding the isolation of all the lipogenic enzymes in the form of a complete multienzyme complex from any source.

We report here the isolation and identification of a lipogenic multienzyme complex (lipid metabolon) from an oleaginous fungus, *Cunninghamella bainieri* 2A1 isolated from Malaysian soil^21,22^, using Blue Native PAGE (BN-PAGE) coupled to liquid chromatography-tandem mass spectrometry (LC-MS/MS). We also provide evidence suggesting the involvement of the formation and dissociation of the multienzyme complex in the regulation of lipid biosynthesis. It was essential that minimal force was used to disrupt the cells in order to preserve the intracellular structures. This was achieved by preparing protoplasts that were then osmotically lysed. Other direct methods of cell disruption were too severe and always led to the loss of metabolon structures (unpublished work).

## Results

### Detection of lipogenic multienzyme complex using BN-PAGE coupled with LC-MS/MS

**Figure 2:**
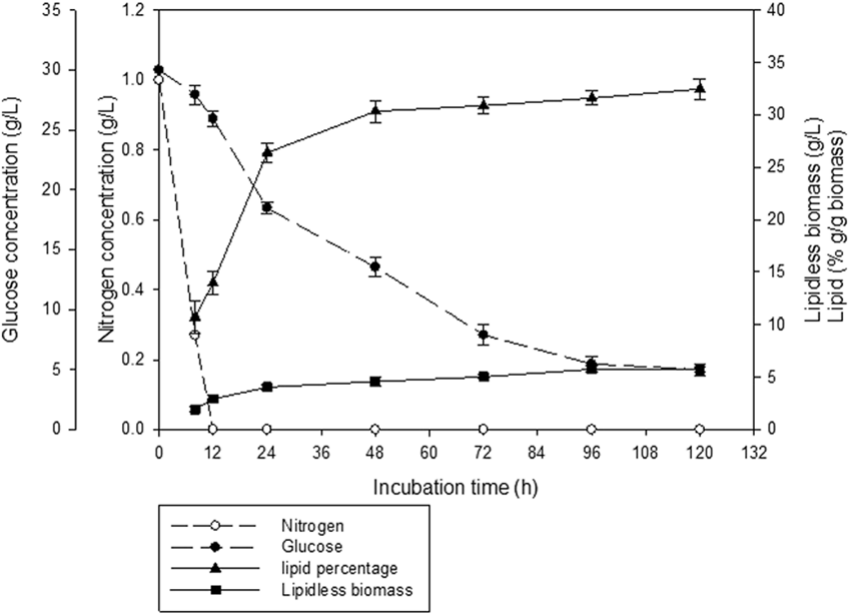
Profiles of growth and lipid accumulation in *C*. *bainieri* 2A1 cultivated in 500 mL shake flask containing 200 mL of nitrogen-limited medium for 120 h, 200 rpm at 30 °C. Error bars based on three biological independent experiments.

Figure 2 shows the profiles of growth and lipid accumulation of *C*. *bainieri* 2A1 where phases of growth, lipid accumulation and cessation of lipid accumulation occurred between 0 to 8 h, 24 to 48 h and 96 to 120 h, respectively.

**Figure 3:**
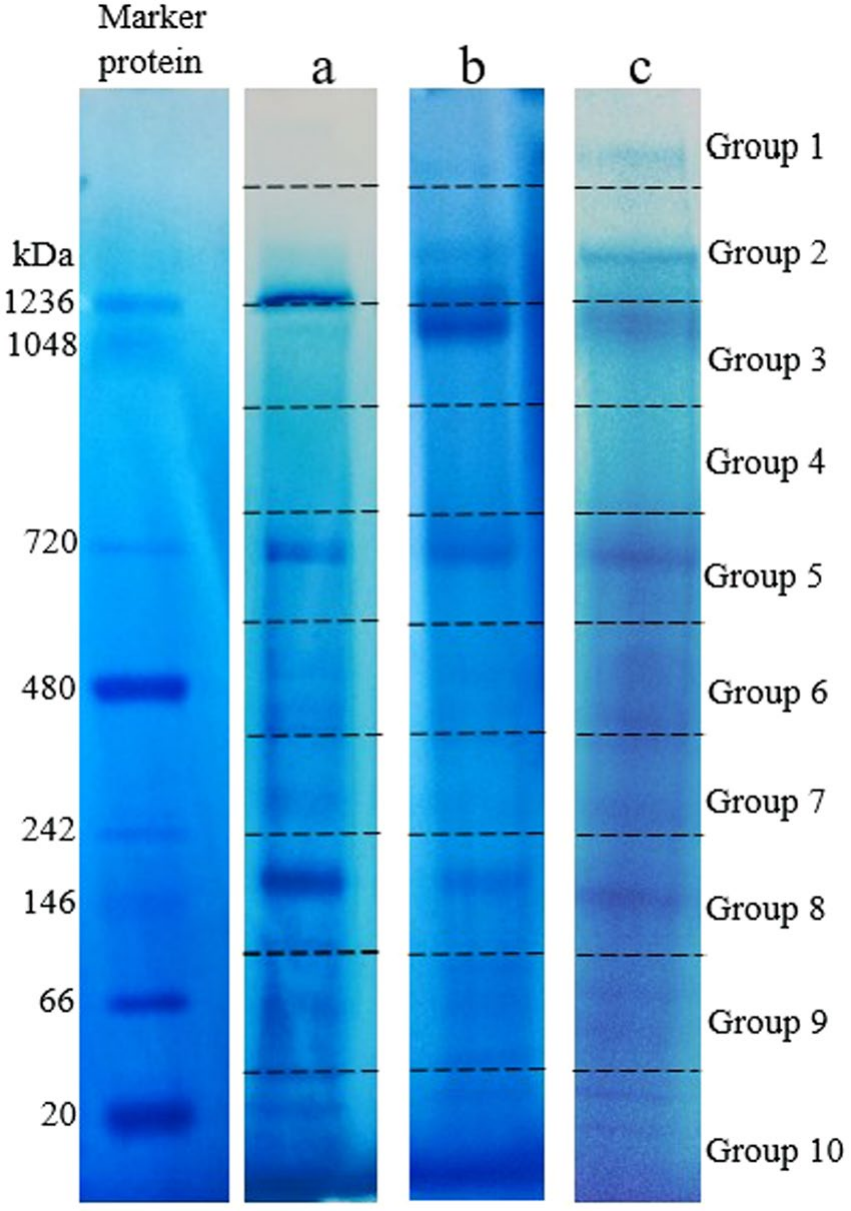
BN-PAGE of the crude cell extract obtained at various cultivation intervals of *C*. *bainieri* 2A1. (**a**) Culture of 8 h (balanced growth phase). (**b**) Culture of 24 h (the beginning of lipid accumulation). (**c**) Culture of 96 h (after cessation of lipid accumulation phase). After staining with Coomassie brilliant blue G-250, the gels were cut into 10 slices with the measurement approximately of 8 × 8 mm per gel slices. Each slice was designated as group 1 (upper part of the gel) until group 10 (lowest part of the gel). All gels for BN-PAGE were run under the same experimental conditions. Shown is the cropped gel (The gel with indicated cropping lines is shown in Supplementary Figure S1a–c).

Figure 3a–c represent BN-PAGE of the extracts obtained from the 8 h (balanced growth phase), 24 h (lipid accumulation phase) and 96 h (after cessation of lipid accumulation phase) cultures of *C*. *bainieri* 2A1. Proteins in a cell extract obtained by disruption of protoplasts prepared from these phases were separated by BN-PAGE gradient gel (3–12% providing resolutions of 15 kDa to 10 MDa). The approximate molecular weights of the proteins were estimated based on exponential plot of molecular weight of standard proteins against relative migration distance (R_f_) of standard proteins.

After the electrophoresis, the gel was sliced into 10 groups with an approximate length of 8 mm each, and designated as group 1 to group 10. Group 1 represents the upper part of the BN gel and group 10 represents the lowest part of the gel. The estimated molecular weight of the proteins that could be resolved in each gel groups are as follows; group 1 (4.6 MDa to 10 MDa), group 2 (1.2 MDa to 4.6 MDa), group 3 (950 kDa to 1.2 MDa), group 4 (830 kDa to 950 kDa), group 5 (650 kDa to 830 kDa), group 6 (400 kDa to 650 kDa), group 7 (245 kDa to 400 kDa), group 8 (130 kDa to 245 kDa), group 9 (30 kDa to 130 kDa) and group 10 (0 to 30 kDa). The lipogenic multienzyme complex is postulated to consist of the key lipogenic enzymes: FAS, ME and ACL as well as ACC, PC and MDH with their approximate molecular weights of 2.6 MDa, 160 kDa, 375 kDa, 252 kDa, 330 kDa and 70 kDa, respectively^5^. Thus, the molecular weight of the complex is estimated to be more than 3.7 MDa assuming a complex of single enzymes. This should, therefore, be resolved in group 1 or group 2 of the gel.

The LC-MS/MS analysis was done for each 8 h, 24 h and 96 h samples after running the BN-PAGE. The experiments were repeated three times and the results were shown to be reproducible. The data from one set of the experiment was used to represent the results. Results showed that all the targeted enzymes (ME, FAS, ACL, ACC, PC and MDH) were identified in gel group 2 from the sample of 24 h of cultivation (during lipid accumulation phase) (as shown in Table 1). The isolation of the six proteins in a single gel group indicates that the enzymes are physically associated during lipid biosynthesis phase. This observation could not have resulted from inadequate resolving time as the electrophoresis was run for 24 h. To the best of the author’s knowledge, this is the first evidence confirming the existence of the proposed lipogenic multienzyme complex consisting of ME, FAS, ACL, ACC, PC and MDH. In addition, each of the enzymes were also resolved in different gel groups representing each individual molecular weight, representing the free forms of each of the enzymes. Results showed that PC, ACL and ACC were present in gel group 7 while ME and MDH were detected in gel group 8 and 9, respectively (as shown in Table 1). This suggests possible partial dissociation of the complex occurred although gentle disruption method was used. This also indicates some probable equilibrium occurring between the metabolon and the free form enzymes. However, this is uncertain as it is unknown how much unravelling of the metabolon might have occurred accidentally during the isolation procedures. Although metabolon is known to be fragile, until better methods have been developed to stabilize it, it is inconclusive whether the detached proteins are there in equilibrium with the metabolon or have arisen as artefacts.

**Table 1:** Targeted enzymes detected in gel group 2, 4, 7, 8 and 9 following sampling of the cultures at 24 h (the beginning of lipid accumulation).

Gel group	Protein	Accession number	Distinct peptide	Peptide score	SPI (%)	Peptide sequence	Peptide coverage(%)	Protein score
2	FAS	S2JBT1	4	10.71 9.05 10.16 12.83 12.95 9.05	81.5 66.8 70.1 65.5 70.0 68.6	APTGLDQSR DIAVLK TFNDTALR YVVDSIAR LAIVDR VVVTTSR	2	64.67
	ME	S2J9V8	2	14.3611 78 10.58 11.51 9.45	77.5 74.3 61.2 76.4 73.4	AELPELCQTIR ILGLGDLGTNGIGIPIGK TLPIILDLGTNNEK QIQSYFQIEHNMTEEEAK HVFWIVDSK	10	57.68
	ACL	S2JM91	7	14.49 10.85 10.11 18.22 17.38 8.24 9.07 18.33 14.11 14.54 17.09 20.35 13.23 17.61 11.02 10.27 8.56	89.1 75.4 64.7 89.9 89.5 67.3 66.1 88.5 80.0 76.4 95.1 91.5 75.1 90.6 73.5 76.6 68.1	LVAKPDQLIK HGLLTLNK QWIEER IDQTAEFEAGPK EEAYIQELDGK TILDLMTR ALTEFK GGPNYQEGLR AFVYGMQPR FYWGTK TIAIIAEGVPER TTDGVYEGVAIGGDR KPASFVSTIVDDR AYDSGMTPR EFVTSMR LIPGIGHK VEEITTSK	15	246.91
	ACC	S2J3C8	1	13.36 11.78 9.34 10.58	75.7 74.3 76.3 76.6	LSEIESR TADYLISESDR ADEDMDDTAFR LGADPIDR	2	57.68
	PC	S2JTP7	5	11.08 14.87 12.38 8.07 9.68 10.88 9.74 10.89 12.58 10.61	65.6 73.2 82.4 62.0 61.5 75.3 76.5 76.9 75.4 70.6	TAHELSMR TVAVFSHEDR HIEVQLLADR NAGTAEFLVDNQNR VTTEDPELNFQPDTGK GFAIQCR ALVEFR TNIPFLQR DAHQSLLATR LGIDAVK	6	110.78
	MDH	S2J7L6	3	10.77 14.38 13.17 15.21 14.91 12.02 12.83	80.0 88.7 84.3 82.9 84.9 62.5 72.7	KPGMTR INAGIVR DLAVAAAK IFGVTTLDIVR IQFGGDEVVK DGAGSATLSMAYAGAR GSTFITEGAK	38	92.39
	40 S Ribosomal protein S0	S2JWG6	2	10.41 8.45 7.25	72.6 71.7 61.5	ADGINLINIGK MTPYIHK HAIGLIYWLLAR	10	26.11
	60 S Ribosomal protein L6	S2JHQ8	2	13.42 7.23 11.58	69.9 62.1 71.4	VNQAYVIATSTK QTAVAAPAK GQFPHNMK	12	32.23
4	PFK	S2K2L9	2	12.39 12.06 11.53 10.81	80.6 75.1 77.4 70.9	IGVLTSGGDAPGMNPCVR GLFTPQAQATLAR AVELTHEVAK TVEFYK	5	46.70
7	ACL	S2JM91	4	12.76 9.76 9.96 11.16 12.23 10.17	61.3 64.4 75.4 64.3 74.5 61.2	EGDYILFTHEGGIEVGDVDAK IDQTAEFEAGPK TILDLMTR TIAIIAEGVPER TTDGVYEGVAIGGDR LIPGIGHK	7	66.04
	ACC	S2J3C8	1	12.55 13.07 13.52	76.4 74.3 75.7	IYLSANSGAR ENYQEDETIR LSNFDIKPCFIENR	2	39.14
	PC	S2JTP7	2	11.52 15.09 12.98 14.52 10.47	65.6 73.2 82.4 75.4 76.9	TAHELSMR IGQYTPVAAYLAQDEVVR SEIDIPVLR YFLSKPEINEEFHVEIEEGK NSALEVVTR	5	64.58
	PK	S2J681	2	13.21 12.37 11.05	78.4 76.5 66.7	TGLMLNDTEVPIK WDEDVESR WGIQQGK	5	36.63
8	ME	S2J9V8	2	11.52 11.78 10.59 14.36	76.4 74.3 61.2 77.5	SGYLNEGK AELPELCQTIR GDNEGQQYK ALADSLTEEEISK	7	48.52
	G-6-PDH	S2KGV4	3	12.54 11.26 9.17	79.4 71.5 69.8	TYPALFGLYR NVMNLR FANVLFGHAWDR	6	32.97
	NADP^+^: ICDH	I1CKX9	2	13.22 11.68 9.79	78.3 76.9 66.8	LVPGWTNPIIIGR LIDDMVAQALK MPLYLSTK	8	34.69
9	MDH	S2J7L6	2	12.91 11.58 12.14	78.5 69.7 73.2	LSQEQIEK FALNIVEAVVAGK LFSAATSELK	9	36.63
	6-PGDH	S2KIR3	2	14.71 13.22 10.04 9.58	81.8 76.2 75.9 69.1	MVHNGIEYGDMQLICEIYQVMK WTGIDSLDR GILFVGSGVSGGEEGAR ILDTAGQK	12	47.55

However, when extracts of cultures undergoing balanced growth (8 h) and in the phase after cessation of lipid biosynthesis (96 h) were analysed by LC-MS/MS, all the enzymes were detected in gel groups corresponding to their molecular weight (as shown in Table 2 and Table 3, respectively) and no evidence of possible physical association into any aggregate was observed. For both samples, only FAS (2.6 kDa) was detected in gel group 2 whereas PC, ACL and ACC were in gel group 7 while ME and MDH were in gel group 8 and 9, respectively. This suggests that the lipogenic enzymes were not physically associated during growth (8 h) as well as after the conclusion of the lipid accumulation phase (96 h) and that the formation of the lipogenic multienzyme complex occurred transiently, only during lipid accumulation phase.

**Table 2:** Targeted enzymes detected in gel group 2, 7, 8 and 9 following sampling of the cultures at 8 (balanced growth phase).

Gel Group	Protein	Accession number	Distinct peptide	Peptide score	SPI (%)	Peptide sequence	Peptide coverage (%)	Protein score
2	FAS	S2JBT1	3	13.75 11.83 12.74 13.18 11.94	87.8 70.1 81.2 80.6 81.5	GALQNLDALANVLNYIK GHATVPLPGIDVPFHSSFLLSGVTPFR LTSTYLNVLTEIAEQGITFADK DILQESFINTMPAWINMLLLSSSGPIK QWGFQYPLIQVMR	3	63.44
7	ACL	S2JM91	2	12.769.96 11.16 12.23	61.3 75.4 64.3 74.5	EGDYILFTHEGGIEVGDVDAK TILDLMTR TIAIIAEGVPER TTDGVYEGVAIGGDR	5	46.11
	ACC	S2J3C8	1	13.58 13.11 12.59 11.23	75.7 74.3 76.4 76.2	IIEEAPVTIAKPDVFEQMEK ITAENPDAGFK EHITLQSEATAYK ELLILCQLPSYEER		
	PC	S2JTP7	2	11.52 15.21 12.38 9.74 11.37 13.48 10.61	65.6 73.2 82.4 76.5 76.9 75.4 70.6	TAHELSMK TVAVFSHEDR HIEVQLLADR ALVEFR TNIPFLQR DAHQSLLATR LGIDAVK	5	84.31
8	ME	S2J9V8	2	11.58 11.93 12.73 14.52	70.2 76.4 74.3 77.5	LNCTAMDPAK AELPELCQTIR QIQSYFQIEHNMTEEEAK VIFASGTAFPAYTIK	9	50.76
9	MDH	S2J7L6	1	12.12 12.91	73.2 78.5	IFGVTTLDIVR IQFGGDEVVK	1	20.13

**Table 3:** Targeted enzymes detected in gel group 2, 7, 8 and 9 following sampling of the cultures at 96 (after cessation of lipid accumulation phase).

Gel Group	Protein	Accession number	Distinct peptide	Peptide score	SPI (%)	Peptide sequence	Peptide coverage (%)	Protein score
2	FAS	S2JBT1	2	10.71 12.83 12.95 13.86	81.5 67.5 70.9 88.3	IEGALLIAITMEPAAR EFGQALIENCVDVLAESPVYK IMIAGATEDITEESSYEFANMK STLQNEILGDLQK	2	50.35
7	ACL	S2JM91	4	12.76 12.23 11.16 10.21 9.96 9.76	61.3 74.5 64.3 61.2 75.4 64.4	EGDYILFTHEGGIEVGDVDAK TTDGVYEGVAIGGDR TIAIIAEGVPER EGDYILFTHEGGIEVGDVDAK TILDLMTR TIAIIAEGVPEK	8	66.08
	ACC	S2J3C8	1	10.58 9.35 11.78 13.36	76.4 77.8 74.3 75.7	ANAEYIR DEVQATR VEVYQEVK IYLSANSGAR	1	40.07
	PC	S2JTP7	3	11.08 9.74 10.51 10.83 12.31 12.47 14.86	65.6 77.5 70.6 76.9 82.4 75.5 73.2	TAHELSMR DCSVQR HYFIEINPR LVQFQNR SEIDIPVLR LLYSCFEANVR METVVTSPVAGK	7	81.81
8	ME	S2J9V8	2	11.58 11.93 12.73 14.52	76.4 77.5 61.2 74.3	YILMAQLR AELPELCQTIR NYRPTDTEGFEPEIAVISDGK IVFYGAGSAAIGVAR	6	47.73
9	MDH		2	9.98 15.21 11.18 13.03 12.83	88.7 82.9 84.9 62.5 72.7	DLAVAAAK QFNVYDPR IQFGGDEVVK DGAGSATLSMAYAGAR GSTFITEGAK	18	62.23

With regards to other NADPH-generating enzymes (NADP^+^: ICDH, 6-PGDH and G-6-PDH), none of these were found to be associated to the complex detected in gel group 2 during lipid accumulation phase (24 h) where NADP^+^: ICDH and G-6-PDH were detected in gel group 8 and 6-PGDH was detected in gel group 9 (as shown in Table 1). This provides further evidence regarding the exclusive role of ME in the provision of NADPH to FAS. Furthermore, none of the enzymes that served as negative controls [phosphofructokinase (PFK) and pyruvate kinase (PK)] were detected in gel group 2 (as shown in Table 1). The complex that we observed is therefore not a spurious collection of proteins, some involved in lipid biosynthesis and some not, but is a dedicated group of enzymes all with established roles in fatty acid biosynthesis.

**Figure 4:**
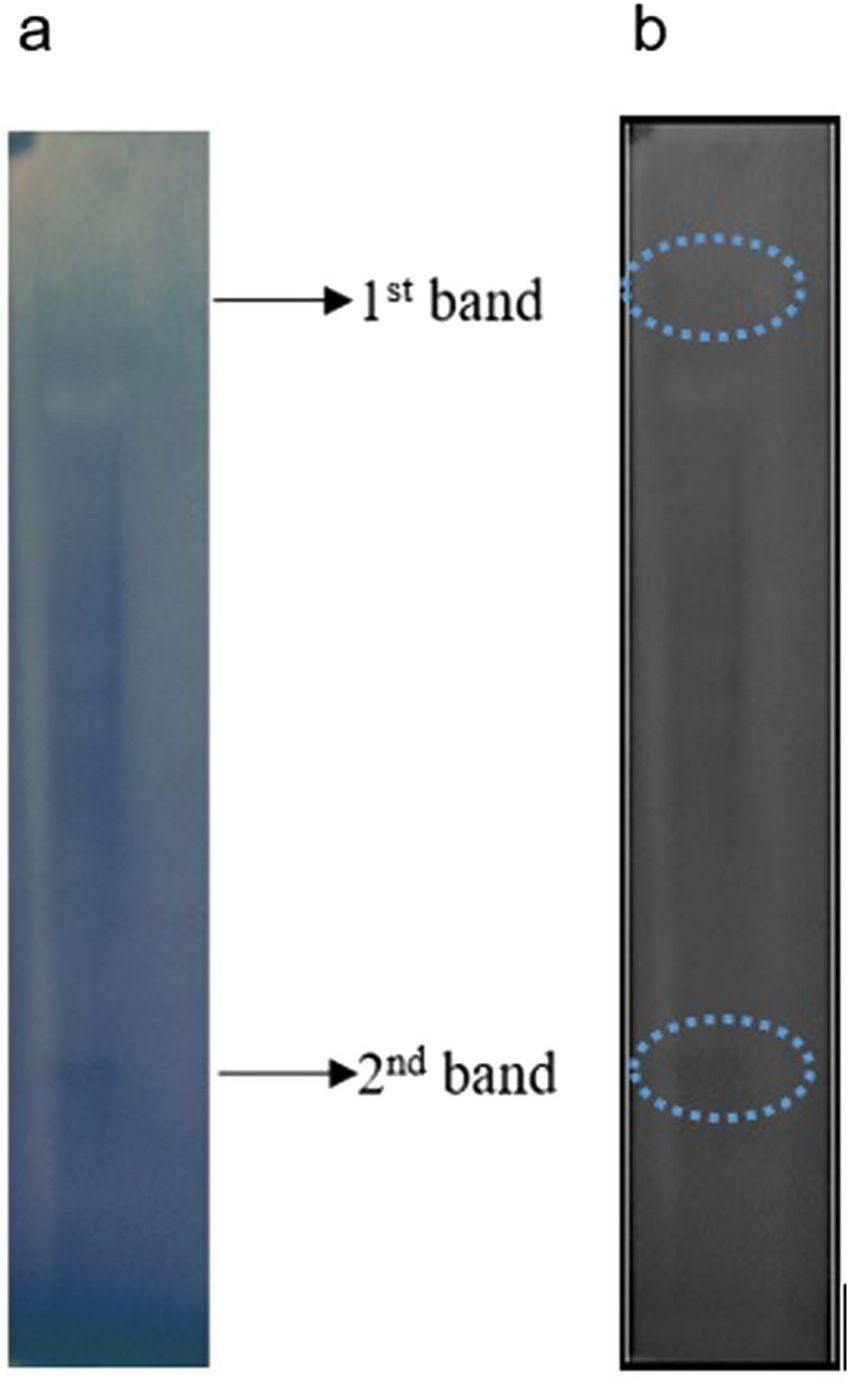
Activity staining of ME on the gradient gel from the culture of 24 h. (**a**) Original picture of the cropped gel with two bands detected. (**b**) Edited picture with the blue background colour removed to highlight the bands. All gels for activity staining of native PAGE were run under the same experimental conditions. Shown is the cropped gel (The gel with indicated cropping lines is shown in Supplementary Figure S2).

The presence of ME in the gel groups 2 and 8 was also confirmed by in-gel activity staining. Results showed that for the 24 h extract, two bands representing ME activity appeared in gel group 2 and 8 (as shown in Fig. 4a and b) whereas for 8 h and 96 h extract, only a single band appeared in gel group 8 thus confirming the tandem mass spectrometry data.

## Discussion

Regulation of lipid accumulation in oleaginous yeasts and fungi has been well established where the extent of lipid accumulation has been reported to be determined by ME^3–5^. The lipid content accumulated by oleaginous fungi correlates with the presence and activity of ME. In *Mcr circinelloides* the specific activity of ME diminished shortly after N-limitation was achieved (18 h cultivation) coinciding with cessation of lipid accumulation with a lipid content of 30% (w/w) although in the presence of other lipogenic and NADPH-generating enzymes. Whereas ME activity of *Mta alpina*, which accumulated up to 45% (w/w) lipid lasted even more than 88 h of cultivation period^9^. A similar observation was also reported in *C*. *bainieri* 2A1^10^. This led to the suggestion that ME plays a specific and crucial role in generating NADPH for FAS catalysis that could be explained only by the existence of physical association between ME and FAS. This would allow direct channelling of NADPH from ME to FAS. Hence a hypothesis was proposed by Ratledge^5^ for the existence of a multienzyme complex involving ME and FAS as well as other essential lipogenic enzymes. Indeed, physical association of several of the enzymes has been reported such as the evidence of physical association between ACL, ACC and FAS in which these enzymes have been successfully isolated as a high molecular weight fraction from rat liver (ME was not included in the work)^19^. The proposed hypothesis involves all lipogenic enzymes catalysing sequential reactions i.e. ACC, ACL and FAS plus ME and MDH and PC which operates as a “transhydrogenase cycle” for NADPH generation. However, up to now, no evidence regarding the existence of the hypothesized complex has been reported. We report the first evidence validating the hypothesis using an oleaginous fungus, *C*. *bainieri*, as a model.

In this work, a lipogenic multienzyme complex consisting of ME, FAS and ACL as well as PC, ACC and MDH in *C*. *bainier*i has been isolated using protoplasts for the initial step followed by their gentle breaking. As we have tried but failed on numerous occasions to isolate this complex starting with whole cells coupled with a variety of cell disruption systems (unpublished work), we argued that harsh cell disintegration was also leading to disruption of any liposome complex. Hence, by using protoplasts, we were able to preserve the internal structure of the cell and this was then kept intact during their gentle lysis. As shown in Table 1, during lipid accumulation (24 h) the enzymes were present in gel group 2 despite significant differences in molecular weight of each individual enzymes indicating positive physical association. The absence of two enzymes (PFK and PK) that serve as negative controls in group 2 indicates the unlikely probability that the observed association could have resulted from non-specific interactions.

The formation of this complex is shown to be transient, as the complex was only detected during lipid accumulation phase (24 h) and was absent during growth (8 h) and after cessation of lipid accumulation (96 h). A similar observation has been reported in *Bacillus subtilis* where a multienzyme complex involving malate dehydrogenase and phosphoenolpyruvate carboxykinase forms only during growth on gluconeogenic carbon sources^17^. In addition, the transient nature of multienzyme complexes was also demonstrated in relation to the association of glycolytic enzymes on the surface of mitochondria in *Arabidopsis thaliana*, where the association was dependent on the rate of respiration. These findings show that the formation of multienzyme complexes can be attributed to the change of metabolic flux where the complex formation would support direct channeling of metabolites for efficient operation of the pathways^23^.

Despite the detection of the lipogenic enzymes as clusters in gel group 2, each of the enzymes was also detected in separate bands corresponding to their molecular weights. This could be due to partial dissociation of the complex during the final disruption of the protoplasts and subsequent handling and possibly during electrophoresis itself. Most multienzyme complexes are fragile and held together by weak interactions that will be strengthened by attachment to appropriate membranes within the cell^24,25^. In this case, we suggest that attachment of the complex to membranes of the endoplasmic reticulum and mitochrondrion (as shown in Fig. 1) might be the key stabilizing factor. Attachment to the membrane of a (growing) lipid droplet is also a possibility. The stability and integrity of multienzyme complexes will also be affected by other factors such as pH, temperature and metal ions concentration^14^ and we make no claims that we have optimized the conditions for the isolation of this liposome complex.

No evidence of involvement of other NADPH-generating enzymes, besides ME, in the lipogenic multienzyme complex was observed. G-6-PDH, 6-PGDH and NADP^+^: ICDH all were not found to be resolved in gel group 2 (as shown in Table 1). This supports the previous suggestion where ME functions exclusively as the NADPH provider for FAS.

## Conclusions

This work provides evidence of the existence of a lipogenic multienzyme complex consisting of ME, FAS, ACL, ACC, PC and MDH. The multienzyme complex exists transiently and was only detected during lipid accumulation phase whilst dissociating during growth and cessation of lipid accumulation. No evidence of probable involvement of G-6-PDH, 6-PGDH and NADP^+^: ICDH was observed indicating the vital role of ME as NADPH provider in lipid biosynthesis in *C*. *bainieri* 2A1.

## Methods

### Microorganism

*Cunninghamella bainieri* was obtained as a stock culture from the School of Biosciences and Biotechnology, Faculty of Science and Technology, Universiti Kebangsaan Malaysia, Bangi, Selangor. The cultures were maintained at 4 °C on potato/dextrose/agar.

### Preparation of media and culture condition

A nitrogen-limited medium^20^ containing 1 g/L diammonium tartrate, 7 g/L KH_2_PO_4_, 2 g/L Na_2_HPO_4_, 1.5 g/L MgSO_4_⋅7H_2_O, 1.5 g/L yeast extract, 0.1 g/L CaCl_2_, 0.008 g/L FeCl_3_⋅6H_2_O, 0.0001 g/L Co(NO_3_)_2_⋅6H_2_O, 0.0001 g/L ZnSO_4_⋅7H_2_O, 0.0001 g/L CuSO_4_⋅5H_2_O and 0.0001 g/L MnSO_4_⋅5H_2_O was sterilized at 121 °C for 40 min. Glucose (to give 30 g/L) was added separately after sterilization. Seed culture was prepared by transferring a spore suspension into 500 mL shake-flasks containing 200 mL nitrogen-limited medium to give 10^5^ spores/mL. The cultures were grown at 30 °C and shaken at 200 rpm for 48 h. Ten percent (v/v) of the culture was then used for subsequent inoculations. All experiments were carried out using 500 mL conical flasks containing 200 mL nitrogen-limited medium as described above. Cultivation was carried out at 30 °C, with agitation at 200 rpm and cultures were harvested at 8 h (growth phase), 24 h (lipid accumulation phase) and 96 h (cessation of lipid accumulation).

### Preparation of protoplasts and cell extract

Mycelia were obtained by filtration through pre-weighed Whatman No. 1 filter paper followed by washing with 400 mL distilled water. 0.075 g of lysing enzymes containing β-glucanase, cellulase, protease and chitinase activities from *Trichoderma harzianum* (Sigma) were added to 12.5 mL stabilizing buffer (1.2 M MgSO_4_ and 10 mM KH_2_PO_4_ at pH 6) and filtered by membrane filter of 0.2 µm. 0.5 g of filtered mycelia were suspended in this stabilizing solution (containing the lysing enzymes) and the mixture was held for 3 h at 30 °C with gentle shaking (100 rpm). Mycelia debris was removed by filtration through 3 layers of lens-cleaning tissue^26,27^. The protoplasts were observed and counted using Neubaeur hemacytometer and then centrifuged at 2000 x *g* at 4 °C for 10 min. The pellet (protoplasts) was suspended in 5 mL extraction buffer (0.1 M KH_2_PO_4_, 20% (v/v) glycerol, 1 mM benzamidine, 1 mM mercaptoethanol and 1 mM EDTA) for 15 min to lyse the protoplasts, centrifuged at 10,000 x *g* for 15 min at 4 °C and the supernatant (cell extract) obtained was used for subsequent experiments.

### Determination of protein concentration

Protein concentration was measured according to the method of Bradford.

### Blue Native Page Polyacrylamide Gel Electrophoresis (BN-PAGE)

#### Sample preparation

Cell extract was diluted 6:1 (v/v) with Coomassie Brilliant Blue G-250 (5%; w/v) at 4 °C.

#### Preparation of cathode buffer

Fifty mL of 20x NativePAGE™ running buffer (Invitrogen) and 50 mL 20x NativePAGE™ cathode additive (Invitrogen) were added to 900 mL deionized H_2_O for preparation of 1x NativePAGE™ cathode buffer at pH 7.5 and 4 °C. Cathode buffer consists of 15 mM Bis-Tris, 50 mM trycine and 0.02% (w/v) Coomassie Brilliant Blue G-250.

#### Preparation of anode buffer

Fifty mL of 20x NativePAGE™ running buffer (Invitrogen) was added to 950 mL of deionized H_2_O for preparation of 1x NativePAGE™ anode buffer at pH 7.5 and stored at 4 °C. Anode buffer consists of 50 mM Bis-Tris.

#### Separation of proteins by BN-PAGE

Lipogenic multienzyme complex in crude cell extract was separated by BN-PAGE^28^. Briefly, a precast native gradient gel (Native PAGE™ Novex Bis-Tris Gels, Invitrogen) with 3–12% gel gradient were used. This gel provides molecular weight resolution from 15 kDa to 10 MDa. The BN-PAGE was performed by Xcell *SureLock™* Mini Cell (Invitrogen). A 300 mL of cathode buffer was added into the upper buffer chamber followed by the addition of 600 mL of anode buffer into the lower buffer chamber. Twenty µL of cell extract containing approximately 80 µg protein were loaded to each well. Twenty µL standard protein NativeMark™ unstained protein standard (Invitrogen) consisting of IgM hexamer (1236 kDa), IgM pentamer (1048 kDa), apoferritin band 1 (720 kDa), apoferritin band II (480 kDa), β-phycoerythrin (242 kDa), lactate dehydrogenase (146 kDa), bovine serum albumin (66 kDa) and soy trypsin inhibitor (20 kDa) was loaded into the well of the same gradient gel. All steps were performed at 4 °C. The molecular weight of the proteins that resolved in the gradient gel were estimated based on exponential plot of molecular weight of standard proteins against relative migration distance (R_f_) of standard proteins.

#### Electrophoresis

Electrophoresis was performed at 4 °C, 100 V for 3 h and subsequently run overnight at 150 V.

#### Slicing of the BN-PAGE gel

After the electrophoresis, the gel was sliced into 10 groups with an approximate length of 8 mm each, and designated as group 1 (upper part of the gel) to group 10 (lowest part of the gel).

#### In-gel trypsin digestion

In-gel trypsin digestion^29^ was performed using trypsin profile IGD kit for in-gel digests (Sigma-Aldrich). Each of the gel bands (1 to 10) was further sliced separately into smaller pieces and transferred into silicone tube. Two hundred µL of destaining solution (400 mM NH_4_HCO_3_ in 40% acetonitrile) was added into the tubes and incubated at 37 °C for 30 min. This step was repeated twice.

Reduction of disulphite bonds of the proteins was done by the addition of 200 µL 10 mM dithiothreitol (DTT) with incubation at room temperature for 30 min. The DTT solution was then removed. Two hundred µL 55 mM iodoacetamide was then added into the silicone tubes for alkylation of the proteins and incubated in the dark at room temperature. The solution was then removed and replaced with 400 µL of washing solution (50 mM NH_4_HCO_3_) and incubated at room temperature for 15 min. This step was repeated twice. Then, 400 µl acetonitrile (100%) was added into the silicone tubes to dehydrate the gel pieces and incubated for 10 min at room temperature. The supernatant was removed and the gel pieces were dried by incubation at 50 °C for 5 min.

Next, 20 µL (0.4 µg trypsin) trypsin (1 mM HCl and 40 mM NH_4_HCO_3_ in 9% of acetonitrile) was added into the silicone tube and incubated at room temperature for 5 min. Then 50 µL of 40 mM NH_4_HCO_3_ in 9% acetonitrile was added in the tubes and incubated overnight at 37 °C. The digested peptides were then transferred into new silicone tubes and dried again in vacuum centrifuge. The dried peptides were stored at −20 °C before tandem mass spectrometry analysis was performed.

### Identification of protein using high performance liquid chromatography-tandem mass spectrometry (LC-MS/MS)

Ten uL 0.1% formic acid was added into the dried peptides tubes and centrifuged at 13,000 x *g* for 5 min. Five µL of the supernatant was transferred into new silicone tubes. Samples were analysed by Agilent 6520 Accurate Mass Q-TOF LC/MS-Nano ESI (ChipCube) (Agilent Technologies). The parameters of analysis were as follows: two columns were used to separate the peptides; 1. large capacity chip, C18, 300 Å, 160 nL enrichment column, 2. analytical column, 75 µm × 150 mm (Agilent Part no: G4240-62010), flow rate: 4 uL/min from Agilent 1200 Series capillary pump and 0.3 uL/min from Agilent 1200 Series nano pump, solvents: 0.1% formic acid in water (A); 90% acetonitrile in water with 0.1% formic acid (B), injection volume: 2 uL. Mass spectrometry was operated in positive ion mode with acquired 4 MS spectra s^−1^ from 300 until 3000 *m/z*. Auto MS/MS mode applied from 50 until 3000 *m/z* with total maximum precursor of 4 in a cycle and exception of 2 spectra for 1 minute. Identification of peptides was performed by Spectrum Mill MS Proteomics Workbench (Rev B.04.00.127; Agilent Technologies). Cysteine carbamidomethylation was set as fix and variable modification. Search was done online for *Mucorales* at UniProtKB/Swiss-Prot database. Standard criteria for actual proteins selection were as follows; peptides filtered with score ≥ 8, score peak intensity percentage (SPI%) ≥ 60 and protein filtered with score ≥ 10, mass MH^+^ Error ≤ 10 ppm, local false discovery rate ≤ 0.1% and database Fwd-Rev score ≥ 2.

### In-gel activity staining of ME

Activity staining for ME in gradient gel was performed by immersing the gel in a phosphate buffer (pH 7.4) containing 0.47 mM NADP^+^, 17.2 mM L-malate, 0.1 M MgSO_4_, 0.55 mg/mL nitroblue tetrazolium and 0.097 mg/mL phenazine methosulphate^30^. After 3 h of incubation, the reaction was stopped by replacing the staining solution with 5% (v/v) acetic acid.

### Data availability

All data generated or analysed during this study are included in this published article.

## Electronic supplementary material

Supplementary Information
