# Supplementary material for: First evidence for a multienzyme complex of lipid biosynthesis pathway enzymes in Cunninghamella bainieri
Source: Sci Rep. 2018 Feb 15;8:3077. doi: 10.1038/s41598-018-21452-4 (PMC5814418; doi:10.1038/s41598-018-21452-4)
Supplement: Supplementary file 1 — Supplementary Information [file 41598_2018_21452_MOESM1_ESM.docx]

**First evidence for a multienzyme complex of lipid biosynthesis pathway enzymes in *Cunninghamella bainieri***

**Shuwahida Shuib^1^, Izyanti Ibrahim^1^, Mukram Mohamed Mackeen^2,3^, Colin Ratledge^4^, and Aidil Abdul Hamid^1,*^**

^1^School of Biosciences and Biotechnology, Faculty of Science and Technology, Universiti Kebangsaan Malaysia, 43600 UKM Bangi, Selangor, Malaysia, ^2^School of Chemical Sciences and Food Technology, Faculty of Science and Technology, Universiti Kebangsaan Malaysia, 43600 UKM Bangi, Selangor, Malaysia, ^3^Institute of Systems Biology, Universiti Kebangsaan Malaysia, 43600 UKM Bangi, Selangor, Malaysia, ^4^Department of Biological Sciences, University of Hull, HU6 RX, United Kingdom.

^*^To whom correspondence should be addressed. Email: [aidilmikrob@gmail.com](mailto:aidilmikrob@gmail.com)

^+^these authors contributed equally to this work.

**Supplementary Figures**


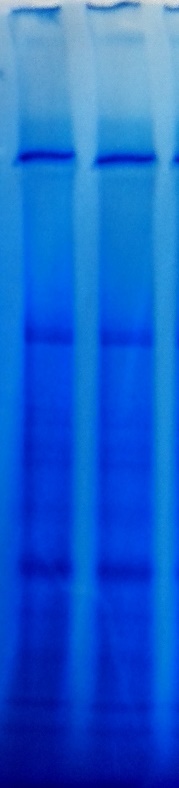


**Figure S1a |** Gel of BN-PAGE. The crude cell extract obtained from the culture of 8 h (balanced growth phase) of *C. bainieri* 2A1.


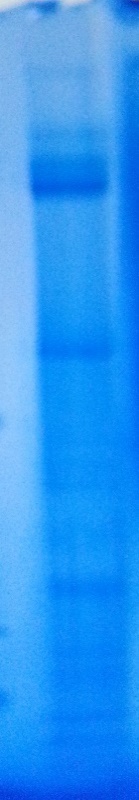


**Figure S1b |** Gel of BN-PAGE. The crude cell extract obtained from the culture of 24 h (the beginning of lipid accumulation) of *C. bainieri* 2A1.


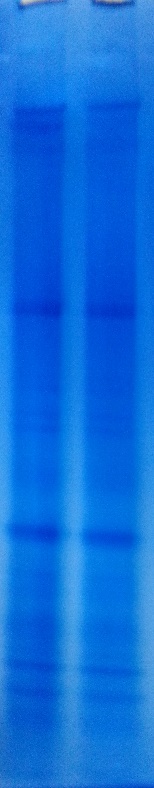


**Figure S1c |** Gel of BN-PAGE. The crude cell extract obtained from the culture of 96 h (after cessation of lipid accumulation phase) of *C. bainieri* 2A1.


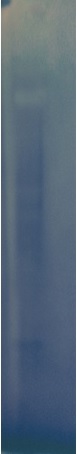


**Figure S2 |** Gel of activity staining of ME on the gradient gel. The crude cell extract obtained from the culture of 24 h.
